# Penetrating Ballistic-Like Brain Injury Leads to MicroRNA Dysregulation, BACE1 Upregulation, and Amyloid Precursor Protein Loss in Lesioned Rat Brain Tissues

**DOI:** 10.3389/fnins.2020.00915

**Published:** 2020-09-18

**Authors:** Bharani Thangavelu, Bernard S. Wilfred, David Johnson, Janice S. Gilsdorf, Deborah A. Shear, Angela M. Boutté

**Affiliations:** ^1^Brain Trauma Neuroprotection Branch, Center for Military Psychiatry and Neuroscience, Walter Reed Army Institute of Research, Silver Spring, MD, United States; ^2^Department of Pathology and Area Laboratory Services, Landstuhl Regional Medical Center, Landstuhl, Germany

**Keywords:** traumatic brain injury, neurodegenerative diseases, microRNA, amyloid precursor protein, beta-site amyloid precursor protein cleaving enzyme 1

## Abstract

Severe traumatic brain injury (TBI) is a risk factor for neurodegenerative diseases. Yet, the molecular events involving dysregulated miRNAs that may be associated with protein degradation in the brain remains elusive. Quantitation of more than 800 miRNAs was conducted using rat ipsilateral coronal brain tissues collected 1, 3, or 7 days after penetrating ballistic-like brain injury (PBBI). As a control for each time-point, Sham-operated animals received craniotomy alone. Microarray and systems biology analysis indicated that the amplitude and complexity of miRNAs affected were greatest 7 day after PBBI. Arrays and Q-PCR inferred that dysregulation of miR-135a, miR-328, miR-29c, and miR-21 were associated with altered levels of beta-site amyloid precursor protein cleaving enzyme 1 (BACE1), PSEN1, PSEN2, and amyloid precursor protein (APP) genes. These events were followed by increased levels of mature BACE1 protein and concomitant loss of full length APP within 3–7 days, then elevation of amyloid beta (Aβ)-40 7 days after PBBI. This study indicates that miRNA arrays, coupled with systems biology, may be used to guide study design prior validation of miRNA dysregulation. Associative analysis of miRNAs, mRNAs, and proteins within a proposed pathway are poised for further validation as biomarkers and therapeutic targets relevant to TBI-induced APP loss and subsequent Aβ peptide generation during neurodegeneration.

## Introduction

Severe traumatic brain injury (TBI) remains a significant health issue that leads to cognitive and physical impairment, prolonged hospitalization, and the need for long-term care (Langlois et al., 2005; Zaloshnja et al., 2008; Cuthbert et al., 2015; Taylor et al., 2017). Military personnel are at a particularly high risk for encountering a TBI (Santiago et al., 2012) due, in part, to improvised explosive devices and munition exposures (Meyer et al., 2010; Boutte et al., 2019). Patients who suffer from a TBI are at a much higher risk of developing neurodegenerative disease or dementia (Plassman et al., 2000), particularly Alzheimer’s disease (AD) (Mendez et al., 2015), Parkinson’s disease (PD) (Gardner et al., 2018), Amyotrophic lateral sclerosis (ALS) (Franz et al., 2019), psychological disorders (Veitch et al., 2013), post-traumatic stress disorder (PTSD) (Friedemann-Sanchez et al., 2008), and suicide (Goldstein and Diaz-Arrastia, 2018). The epidemiological basis for TBI as a risk factor for neurodegeneration is established, yet the molecular basis that may offer a connection between these two events remains difficult to discern. As such, development of effective, possibly preventative, therapeutic strategies remain challenging.

MicroRNAs (miRNAs), small, non-coding nucleotide sequences, approximately 18–25 nucleotides in length that play an integral role in the regulation of gene expression (Fabian et al., 2010). MiRNAs are implicated in neurodegenerative disorders (Eacker et al., 2009) and linked to increased cell death after brain injury (Truettner et al., 2013). A single miRNA is capable of regulating dozens, if not hundreds, of mRNA transcripts, or several miRNAs may be associated with key processes, thereby affecting a myriad of cellular processes, including inflammation (Khoshnam et al., 2017), cell death (Boone et al., 2017), as well as protein turnover or loss (Che et al., 2017) through direct or indirect interactions. Recent studies have indicated that miRNA levels are dysregulated in a host of neurodegenerative diseases and contribute to pathology (Nelson et al., 2008; Maciotta et al., 2013). A large number of miRNAs are associated with amyloid precursor protein (APP) degradation and subsequent amyloid beta (Aβ) production (Schonrock et al., 2012), a pathological hallmark of amyloidopathies and neuronal loss (Karnati et al., 2015; He et al., 2018). TBI leads to widespread atrophy that is spurred, in part, by protein degradation, inclusive of full length APP loss and increased levels of Aβ peptides (Purushothuman et al., 2013). Thus, it is possible that miRNAs may have a role as key mediators of trauma induced neurodegeneration associated with degradation of full length APP in the brain.

In the present study, we determined time-dependent quantitation profiles of miRNAs within ipsilateral rat brain tissues collected 1–7 days after penetrating ballistic-like brain injury (PBBI), a model of severe, open skull TBI. This model has been extensively characterized; several studies have found that key features are generally highest at 1, 3, or 7 days post-injury. For instance, blood brain barrier disruption is greatest within 1 day (Cunningham et al., 2014), while indices of inflammation, cell loss, and protein fragmentation are prominent at 3–7 days post-injury (Boutte et al., 2016, 2020; Cartagena et al., 2016; DeDominicis et al., 2018). Collectively, these features are involved in severe TBI progression for which miRNA may be involved.

Compared to controls, miRNAs were associated with well-known effects of brain trauma, such as inflammation or neurodegeneration. A subset of miRNAs mapped specifically to genes within the APP-processing pathway, including beta-site amyloid precursor protein cleaving enzyme 1 (BACE1), the gene that encodes beta (β)-secretase. These observations were associated with increased levels of mature BACE1, post-transcriptional APP loss, and upregulation of Aβ-40 peptide levels in the PBBI model. Analysis of miRNA arrays and pathway mapping augment hypotheses regarding molecular events of TBI progression. More importantly, subsets of miRNAs, mRNAs, and proteins within a concerted pathway, such as APP processing, may provide novel therapeutic targets for TBI.

## Materials and Methods

### Surgical Procedures

Male Sprague–Dawley rats weighing 250–300 g (Charles River Labs, Raleigh, VA, United States) were housed individually under a normal 12-h light/dark cycle (lights on at 6:00 am). Animals were anesthetized with 5% isoflurane delivered along with oxygen for surgery while body temperature was maintained at 37°C using a heating blanket (Harvard Apparatus, Holliston, MA, United States). Prior to euthanasia for bio-specimen collection, animals were anesthetized with 70 mg/kg ketamine and 6 mg/kg xylazine. Facilities at the Walter Reed Army Institute of Research (WRAIR) are accredited by the Association for Assessment and Accreditation of Laboratory Animal Care International (AAALAC). The experimental procedures were approved by the WRAIR Animal Care and Use Committee. Research was conducted in compliance with the Animal Welfare Act and other federal statutes and regulations relating to animals and experiments involving animals and adheres to principles stated in the Guide for the Care and Use of Laboratory Animals, NRC Publication, 2011 edition.

### Penetrating Ballistic-Like Brain Injury Rodent Model and Brain Tissue Collection

The rodent PBBI model of severe TBI has been extensively characterized (Williams et al., 2005, 2006). The injury trajectory produces a cavity in the brain, mimicking the trajectory of a high-velocity bullet wound. This penetrating brain injury was performed as previously reported (Boutte et al., 2016). Briefly, induction of a 10% (brain volume) unilateral frontal PBBI was performed upon anesthetized rats by stereotaxic insertion of a specially designed probe into the right hemisphere of the brain (Mitre Corporation, McLean, VA, United States). The probe was inserted through a cranial window over the frontal cortex and rapid inflation/deflation of a water-filled balloon was used to create a temporary cavity in the cerebrum. Sham-operated rats received identical surgical procedures and craniotomy, without probe insertion or balloon inflation. Two millimeter thick coronal sections were dissected starting at 5 millimeters from bregma collected 1, 3, or 7 days after PBBI or Sham-operated procedures (*N* = 10/group/time-point). The ipsilateral hemisphere was immediately flash frozen in liquid nitrogen, and stored at −80°C until use. Ipsilateral tissues are comprised of the injury core and perilesional injury zones of the cortical and subcortical regions, including the frontal cortex caudate-putamen as well as the corpus callosum.

### RNA Isolation and Reverse Transcription

Total RNA was isolated from brain tissue samples with the mirVana RNA isolation kit, according to the manufacturer’s protocol specifically modified for total RNA (Ambion Inc., Mississauga, ON, Canada). Content and quality was determined with the Nanodrop 1000 spectrophotometer (Thermo Scientific, Inc., Waltham, MA, United States). Samples were then stored at −80°C until use for miRNA arrays or individual PCR of specific miRNAs or mRNAs.

### MiRNA Arrays

MicroRNAs arrays were conducted as described by the Genetic Resources Core Facility at Johns Hopkins University using methodology provided directly by the manufacturer (Applied Biosystems of Thermo Fisher, Grand Island, NY, United States) as described (Grigorenko et al., 2011). Complementary (c) DNA for 821 target genes for the rodent miRNAs were prepared using MegaPlex^®^ rodent primer pools A and B and arrays were conducted using the TaqMan Rodent Array Panel on the 12-Flex Open Array system (#4461105, Life Technologies/Thermo Fisher, Grand Island, NY, United States). Global normalization was applied prior to further analysis (Mestdagh et al., 2009). Thereafter, the values of PBBI cohorts were compared to respective Sham-operated values to derive the ΔΔCt values. The derived relative quantitative values are reported using the formula for relative quantities RQ = 2^–ΔΔCt^. For each time-point, comparisons were constructed and plotted as −log_2_ fold change of PBBI versus Sham-operated [−log_2_ (PBBI/Sham)] compared to the −log_10_ (*p*-value) with the Quant Studio12K^TM^ software v1.2.2 (Life Technologies/Thermo Fisher, Grand Island, NY, United States). The dataset is available within Supplementary Tables 1A–C.

### Quantitative PCR Assays

Coding (c) DNA was generated using TaqMan miRNA Reverse Transcription primers (16°C for 30 min, 42°C for 30 min, 85°C for 5 min) specific for each miRNA or mRNA sequence. Each sample was tested in duplicate using the AB7500 Fast RT-PCR system (Life Technologies/Thermo Fisher, Grand Island, NY, United States). Relative quantities were calculated using the RQ = 2^−*Ct*^ method with mammU6 or β-actin as the endogenous reference genes for miRNA or mRNA, respectively. The Taqman assay identification numbers (IDs) for the miRNA or mRNA targets are listed as follows: miR-29c (TM000587), miR-328 (TM000543), and miR-135a (TM000460), miR-21 (TM000397, miR-21-5p) and miR-214 (TM000517), mammU6 (TM001973), APP (Rn01524846_m1), BACE1 (Rn00569988_m1), PSEN1 (Rn00569763_m1), PSEN2 (Rn00579412_m1), β-actin (Rn00667869_m1). For each time point, the PBBI values are displayed as a fold change (mean ± SEM) from Sham-operated normalized to “1.”

### Western Blotting

Brain tissues were sonicated for 10 s, three times each, in ice-chilled 1× RIPA lysis buffer, supplemented at 1/100 with Halt protease and phosphatase inhibitor mix (Sigma-Aldrich, St. Louis, MO, United States), followed by centrifugation at 10,000 × *g* at 4°C for 20 min. Clarified supernatant was collected and protein concentrations were determined using the Pierce Micro BCA protein assay kit (Thermo Fisher Scientific, Rockford, IL, United States). Samples were normalized to contain 5 μg total protein/5 μL/lane prior to being separated by 4–15% gradient PAGE with the NuPAGE system (Invitrogen of Thermo Fisher, Grand Island, NY, United States) (Eaton et al., 2013). After transferring to nitrocellulose membranes, blots were probed with anti-C-terminal APP antibody (#A8717, Sigma, Allentown, PA, United States), or anti-BACE1 antibody (clone EPR19523, #ab183612, Abcam, Cambridge, MA, United States). Densitometry of protein bands intensity was measured using the Li-COR Odyssey^®^ CLx Imaging System (Li-COR Biosciences, Lincoln, NE, United States). Contrast was enhanced for visualization purposes only. For each time point, the PBBI values are displayed as a fold change from Sham-operated, which are normalized to equal “1.” Data is displayed as the fold change (mean ± SEM).

### Electrochemiluminescent ELISAs

Clarified supernatant containing 20 μg of total protein was used to determine Aβ-40 and -42 peptide concentrations using the V-PLEX Kit (4G8-epitope) according to the manufacturer’s instructions (Mesoscale, Rockville, MD, United States). Measurements were derived from electrochemiluminescent signal with a QuickPlex SQ120 (Meso Scale Discovery, Rockville, MD, United States) and quantitation was extrapolated from individual standard curves of each peptide. All assays were conducted in duplicate and RIPA lysis buffer was used as a negative control.

### Data Management and Statistical Analysis

Array generated data was analyzed using QuantStudio Expression Suite^®^ software package (Version 1.1) which calculates fold changes and *p*-values of each dataset at each time-point. MiRNA was mapped to direct interactions within the APP processing pathway using Pathways Studio Web with the Fisher’s exact test (Version 12, Elsevier, Radarweg, Amsterdam, Netherlands). Relationships evidenced by at least one citation were retained. Static annotations and nomenclature are shown as defined by Pathway Studios. Aβ peptides were noted as “proteins” by default and have been manually annotated as “peptides” for accuracy. Venn diagram analysis was performed using Venny (Version 2.1)^1^. MiRNA, mRNA, APP, and BACE1 protein levels were analyzed with the two-tailed, Student’s *t*-Tests. Comparative Aβ content were determined using two-way ANOVA, Fisher’s LSD post-test.

## Results

### Temporal miRNA Response in Brain Tissues After PBBI

**FIGURE 1:**
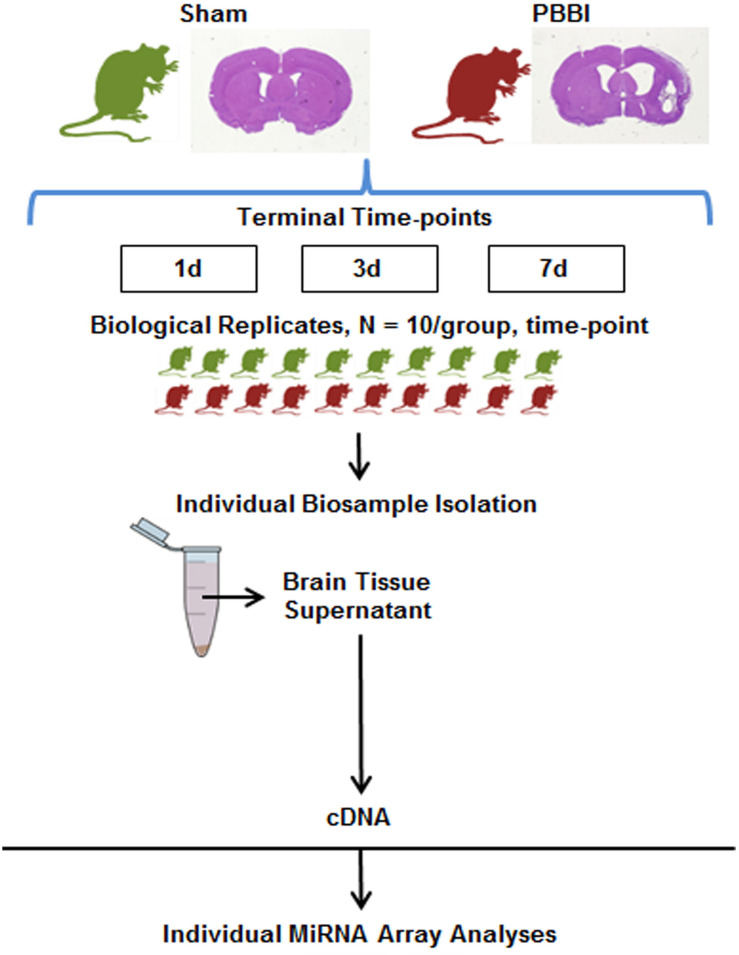
Experimental Design. Craniotomy (Sham-operated) or penetrating ballistic-like brain injury (PBBI) was conducted and ipsilateral, coronal brain tissue containing the injury core and perilesional injury zone which includes contain cortical and subcortical regions of the frontal cortex caudate-putamen and corpus callosum were collected 1, 3, or 7 days post-injury (*N* = 10/group/time point). Total RNA was isolated from with brain tissue supernatant and used to prepare miRNA specific cDNAs. Resulting cDNA was analyzed using the miRNA array containing probes for ∼800 miRNA genes.

**FIGURE 2:**
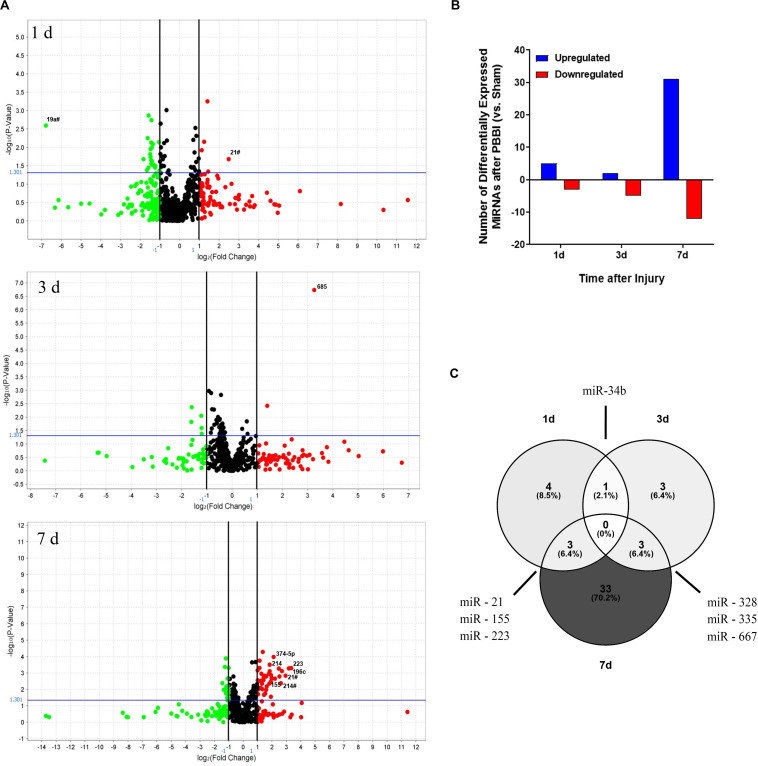
Differential Abundance of MiRNAs after PBBI. Overview of MiRNAs Detected in Brain Tissues after PBBI. **(A)** Volcano plots of miRNA data derived from Life Technologies MiRNA microarray containing ∼821 unique probes. Data is shown for ipsilateral brain tissues collected 1 day (left), 3 day (center), and 7 day (right) after PBBI. All fold change values are normalized to Sham-operated controls. Data is displayed as the Log_2_ ratio of PBBI/Sham (*x*-axis) and the –Log_10_ *p*-value derived from two-tailed *t*-tests (*y*-axis). Black vertical lines represent the Log_2_ (PBBI/Sham) ratio of miRNAs that are decreased (green circles) or increased (red circles). MiRNAs with ≥ two-fold change are annotated. The horizontal blue line represents the –log_10_ *p*-value cut-off at *p* = 0.05. **(B)** Amplitude for each time point tested (*x*-axis), the total number of upregulated (blue) or downregulated (red) miRNAs are displayed (*y*-axis). **(C)** Venn diagram of differentially regulated miRNAs. The total number of differentially expressed miRNAs are indicated for each time-point. Overlap between time-points of specific miRNAs are indicated.

This study examined miRNA levels in coronal brain sections, derived from the ipsilateral hemisphere, at three time points after PBBI. The injury paradigm with the genomics workflow and schema is displayed (Figure 1). The miRNAs affected by injury over the time-points tested were differentiated using miRNA arrays. The effect upon miRNA differential abundance was determined using ipsilateral, coronal brain tissue sections collected after PBBI compared to Sham-operated controls. The number of miRNAs which were differentially abundant varied over time (Figure 2). A total of eight, seven, and 46 miRNAs were differentially expressed at 1 day (Table 1A), 3 day (Table 1B), and 7 day (Table 1C), respectively. Volcano plots indicating the fold changes and *p*-values for each miRNA tested are displayed for each time point (Figure 2A). A total of 58 sequences were found to be differentially affected by PBBI. A major shift in the magnitude of miRNA dysregulated was detected at 7 day after PBBI (31 upregulated, 12 downregulated) (Figure 2B). The vast majority of miRNAs that had a significant fold change were generally unique for each time point with the exception of a few sequences (Figure 2C). MiR-34b increased (1.70-fold) at 1 day, decreased 3 day (0.63-fold), but was similar to Sham-operated levels at 7 day. MiR-328, -335, and -667 levels were unaffected by PBBI at 1 day, yet suppressed at 3–7 days (range: 0.63 to 0.83-fold). Forms of miR-21 was increased at 1 and 7 days after PBBI. Specifically, mmu-miR-21 (miR-21#, miR-21-3p) was increased after 1 day (2.90-fold), while hsa-miR-21 (e.g., miR-21-5p) was increased at 7 day after PBBI (1.50-fold). MiR-223 was also elevated (3.30-fold) at 1 and 7 days. MiR-155 increased within 1 day (3.70-fold), but was slightly less robust (2.2-fold) 7 day after PBBI compared to Sham-operated controls. At 3 day after PBBI, levels of each of these miRNAs was similar to that of Sham-operated controls.

**TABLE 1:** List of miRNAs that were increased or decreased in brain tissue isolated at (A) 1 day, (B) 3 day, and (C) 7 day after PBBI compared to Sham-operated controls over time as defined by Quant Studio.

miRNA Entry Name	Assay ID	Pathway Studio Name	Average Fold Change (vs. Sham)	*p*-Value
**A**				
mmu-miR-19a#	TM002544	miR-19a	0.10	<0.01
mmu-miR-327	TM002481	miR-327	0.59	0.04
hsa-miR-135a	TM000460	miR-135a	0.59	0.04
mmu-miR-21#	TM002493	miR-21	1.20	0.02
mmu-miR-34b-5p	TM002617	miR-34b	1.70	0.05
hsa-miR-223	TM002295	miR-223	3.30	0.01
mmu-miR-1274A^∧^	TM121150	miR-1274A^∧^	3.50	0.03
mmu-miR-155	TM002571	miR-155	3.70	<0.01

**B**				
hsa-miR-328	TM000543	miR-328	0.63	0.02
mmu-miR-34b-3p	TM002618	miR-34b	0.63	<0.01
hsa-miR-335	TM000546	miR-335	0.83	0.04
mmu-miR-329	TM000192	miR-329	0.83	0.03
mmu-miR-667	TM001949	miR-667	0.83	0.01
mmu-miR-451	TM001141	miR-451	1.40	<0.01
mmu-miR-685^∧^	TM001670	miR-685^∧^	3.30	<0.01

**C**				
hsa-miR-411	TM001610	miR-411	0.67	<0.01
mmu-miR-667	TM001949	miR-667	0.71	0.01
mmu-miR-136	TM002511	miR-136	0.77	<0.01
hsa-miR-335#	TM002185	miR-335	0.83	<0.01
mmu-miR-434-3p	TM002604	miR-434	0.83	0.01
hsa-miR-328	TM000543	miR-328	0.83	0.01
hsa-miR-181a	TM000480	miR-181a (A1)	0.83	0.02
mmu-miR-367c	TM002450	miR-376c	0.91	<0.01
hsa-miR-409-3p	TM002332	miR-409	0.91	0.01
mmu-miR-802	TM002029	miR-802	0.91	0.05
hsa-miR-29c	TM000587	miR-29c	0.91	0.03
hsa-miR-139-5p	TM002289	miR-139	0.91	0.03
mmu-miR-298	TM002598	miR-298	1.10	<0.01
rno-miR-466c	TM002067	miR-466c	1.10	<0.01
hsa-miR-130b	TM000456	miR-130b	1.20	<0.01
hsa-miR-146a	TM000468	miR-146a	1.20	0.01
mmu-miR-503#	TM002536	miR-503	1.30	0.05
hsa-miR-224	TM000599	miR-224	1.30	0.01
mmu-miR-18a#	TM002490	miR-18a	1.30	0.01
rno-miR-450	TM001345	miR-450	1.30	<0.01
hsa-miR-363	TM001283	miR-363	1.40	<0.01
hsa-miR-142-3p	TM000464	miR-142	1.40	<0.01
mmu-miR-28#	TM002545	miR-28	1.40	<0.01
mmu-miR-31#	TM002495	miR-31	1.50	0.02
hsa-miR-10a	TM002288	miR-10a	1.50	0.02
hsa-miR-21	TM000397	miR-21	1.50	<0.01
hsa-miR-20b	TM001014	miR-20b	1.50	<0.01
hsa-miR-147b	TM002262	miR-147b	1.60	0.01
mmu-miR-224	TM002553	miR-224	1.60	0.01
hsa-miR-142-5p	TM002248	miR-142	1.60	<0.01
mmu-miR-449	TM002539	miR-449	1.80	<0.01
hsa-miR-18a	TM002422	miR-18a	1.80	<0.01
hsa-miR-200c	TM000505	miR-200c	1.80	<0.01
mmu-miR-449b	TM001667	miR-449b	1.90	0.03
mmu-miR-34c#	TM002584	miR-34c	1.90	<0.01
mmu-miR-199b	TM001131	miR-199b	1.90	<0.01
hsa-miR-199a-3p	TM002304	miR-199a	2.00	<0.01
mmu-miR-374-5p	TM001319	miR-374	2.10	<0.01
mmu-miR-155	TM002571	miR-155	2.20	<0.01
mmu-miR-214	TM002306	miR-214	2.50	<0.01
hsa-miR-214	TM000517	miR-214	2.50	<0.01
hsa-miR-214#	TM002293	miR-214	2.60	<0.01
hsa-miR-223	TM002295	miR-223	2.70	<0.01
mmu-miR-21#	TM002493	miR-21	2.90	<0.01
rno-miR-196c	TM002049	miR-196c	3.20	<0.01
hsa-miR-223	TM000526	miR-223	3.30	<0.01

*The entry name, assay identification number, pathway Studio assigned name, fold change ratio, *p*-value are indicated for each miRNA that increased or decreased after PBBI compared to Sham-operated controls at each time-point. Values that met significance are displayed (*p* ≤ 0.05, two-tailed, Student’s *t*-test, PBBI vs. Sham). Non-regulatory sequences from RNase P RNA or tRNA are indicated with a caret symbol (^∧^).*

### MiRNAs and mRNAs Associated With the Amyloid Precursor Protein Pathway

**FIGURE 3:**
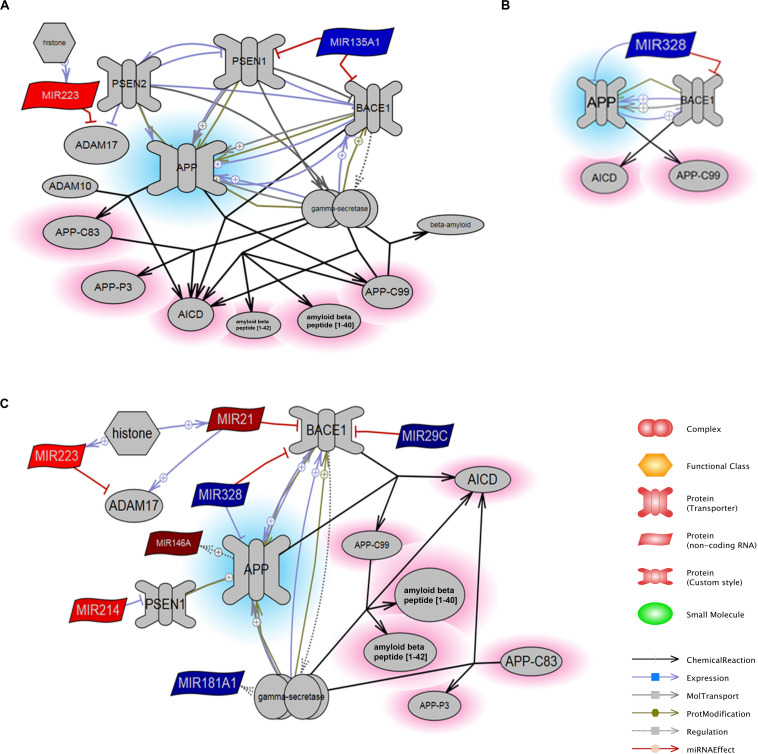
MiRNA Targets of the APP Processing Pathway. The constructed pathways containing miRNAs derived from brain tissues collected at **(A)** 1 day, **(B)** 3 day, and **(C)** 7 day after PBBI are indicated with respective relationships to proteins involved in APP processing. MiRNAs that were decreased (blue) or increased (red) after PBBI compared to Sham-operated controls as derived from microarray results are shown with relationships relevant to APP (blue highlight) and its peptides (red highlight). BACE1 – β-site amyloid precursor protein cleaving enzyme 1, the gene that encode β-secretase; PSEN1/2 – presenilin 1 or 2; APP – amyloid precursor protein, AICD – APP C-terminal fragment produced by γ-secretase cleavage; APP-C99 – APP C-terminal fragment produced by β-secretase encoded by BACE1; APP-C83 – APP C-terminal fragment α-secretase cleavage; APP-P3 – fragment of APP-C83 produced by γ-secretase cleavage. The size of the molecules within images are not to scale with molecular weight or tertiary structure.

Next, this list of miRNAs was mapped to the “APP Processing” pathway in order to determine potential associative relationships to genes involved specifically in APP regulation or degradation, inclusive of down-stream generation of C-terminal fragments and Aβ peptides. The curated interaction as defined by Pathway Studios is shown for each time point (Figures 3A–C). At 1 day, miR-135A (downregulated) was negatively associated with PSEN1 and BACE1 that both converged upon APP via positive relationships (representing cleavage of the target by the enzyme or protease), and note that miR-223 (increased) is displayed with its inhibitory relationship to ADAM17, while ADAM10 has no direct miRNA relationships in this dataset (Figure 3A). Here, regulation of PSEN2 is shown in relation to PSEN1 based on pathways cited from the database. MiR-328 (downregulated) was also linked to BACE1 and APP through negative interactions derived from brain tissues collected at 3 day (Figure 3B). Interactions derived from brain tissues collected 7 day after PBBI indicate that miR-21 (upregulated), miR-29c (downregulated), and miR-328 (downregulated) were also directly linked to BACE1 and APP through inhibitory or inversely proportional relationships defined by Pathways Studio (Figure 3C). MiR-223 (upregulated) was again noted for its negative relationship to ADAM17. Additionally, MiR-214 (upregulated) was negatively associated with PSEN1, which is negatively linked to APP.

**FIGURE 4:**
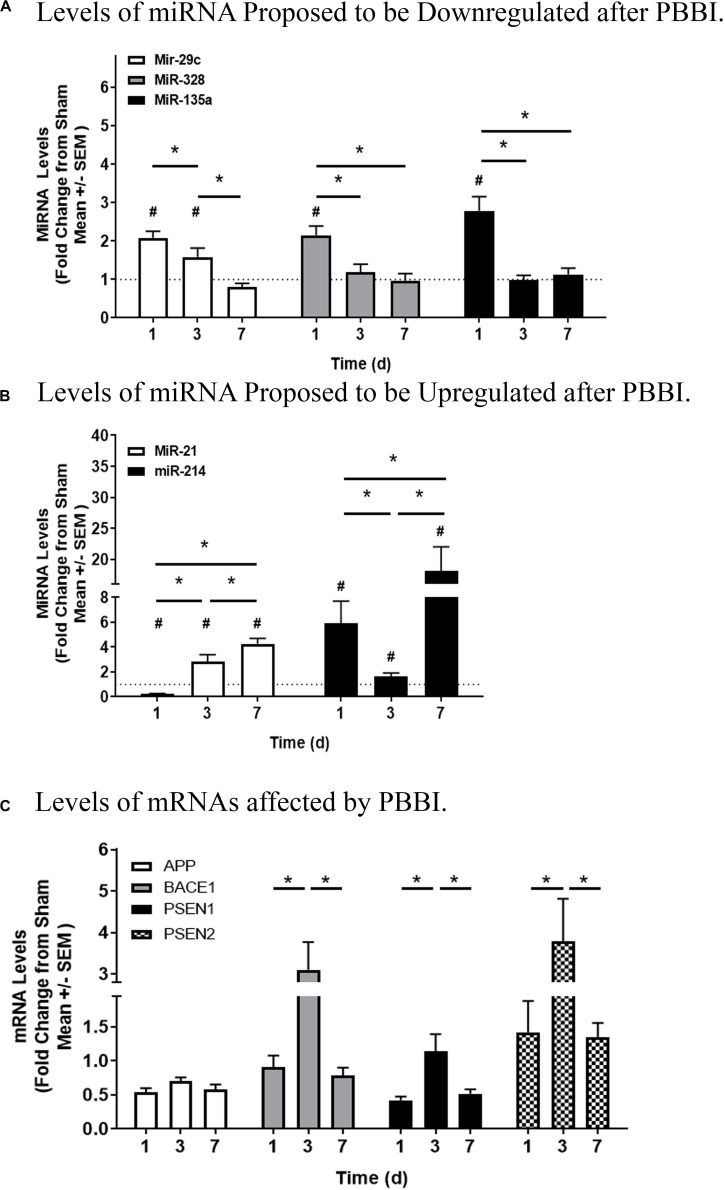
Quantitation of miRNAs and mRNAs Associated with the APP Processing Pathway. Q-PCR of miRNA proposed to be deregulated after PBBI. **(A)** Levels of miRNA proposed to be downregulated after PBBI. MiR-29c (white bars), miR-328 (gray bars), and miR-135a (black bars) are shown. **(B)** Levels of miRNA proposed to be upregulated after PBBI. MiR-21 (white bars) and miR-214 (black bars) are displayed. **(C)** Levels of mRNAs affected by PBBI. APP (white bars), BACE1 (gray bars), PSEN1 (black bars), and PSEN2 (checkered bars) are indicated. MiRNA and mRNA Ct levels were subtracted from Ct values detected for mammU6-RNA or β-actin, respectively. Data is displayed as the fold change (mean ± SEM) normalized Sham-operated control (dotted line = 1) indicating time after injury (*x*-axis) compared to quantitation (*y*-axis). Significant values are defined with an asterisk (#, *p* ≤ 0.05, two-tailed *t*-test, PBBI vs. Sham at each time point; *, *p* ≤ 0.05, PBBI cohorts).

Although array analysis indicated that select miRNAs were significantly dysregulated at specific time points and associated with mRNAs, miRNAs and relevant mRNAs were evaluated across all time points in order to validate array results and to define the temporal effect (Figure 4). Q-PCR indicated that miR-29c, -328, and -135a were each elevated at 1 day (range: 2.08 ± 0.17 to 3.37 ± 0.49), then decreased to near Sham-operated levels 7 day after PBBI (Figure 4A). MiR-21-5p was for single tube, qPCR confirmation. MiR-21 decreased (0.23 ± 0.03) among PBBI groups compared to Sham-operated controls at 1 day, but was progressively increased over time (range: 2.83 ± 0.56 to 4.28 ± 0.43) among PBBI cohorts. Mir-214 was greatest 1 day (5.91 ± 1.80) and at 7 day (18.1 ± 3.92) after PBBI, but slightly less robust at 3 day (1.66 ± 0.25) (Figure 4B). Next, mRNA levels of APP, BACE1, PSEN1, and PSEN2 were determined (Figure 4C). Interestingly, levels of APP mRNA were generally suppressed by PBBI compared to Sham-operated controls (range: 0.53 ± 0.07). Further, BACE1 (3.10 ± 0.11), PSEN1 (1.14 ± 0.25), and PSEN2 (3.79 ± 1.03) mRNA levels each peaked 3 day after injury. PSEN1 was suppressed at 1 day (0.42 ± 0.05) and 7 day (0.52 ± 0.07) compared to Sham-operated controls. However, PSEN2 was similar to Sham-operated levels at these same time points (range: 1.34 ± 0.22 to 1.42 ± 0.46).

### Amyloid Precursor Protein Pathway Protein Levels

**FIGURE 5:**
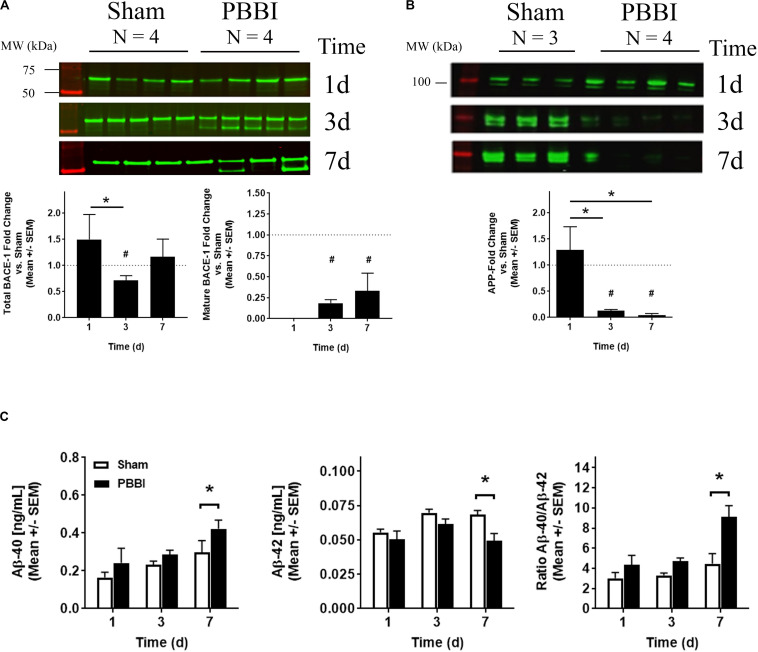
BACE1, APP, and Amyloid β Peptide Quantitation. **(A)** Exemplary western blot images of pro (∼56 kDa) and mature (∼50 kDa) BACE1 is shown (top) with quantitation of either total (bottom left) or mature BACE1 (bottom right). **(B)** Western blot images of APP (100 kDa) (top) and quantitation of APP detected with a C-terminal antibody (bottom) are shown. Data is displayed as the fold change (mean ± SEM) normalized Sham-operated control (dotted line = 1) indicating time after injury (*x*-axis) compared to protein level quantitation (*y*-axis). Significant values are defined with an asterisk (*, *p* ≤ 0.05, 1-way ANOVA; #, Student’s two-tailed, *t*-test vs. Sham-operated control). **(C)** Time dependent (*x*-axis) fold change values derived from Aβ-40 (left), Aβ-42 (center) peptide concentrations, or from the Aβ 40/42 ratio (right) measured by electrochemiluminescent ELISA (mean [pg/mL] ± SEM) are displayed (*y*-axis). Significant values are defined with an asterisk (* *p* ≤ 0.05, two-way ANOVA with Fisher’s LSD post-test).

Due to the increase in BACE1 and suppression of APP mRNA transcripts, protein levels were investigated (Figure 5). Western blotting detected both pro- and mature forms of BACE1 at ∼56 and ∼50 kDa, respectively (Figure 5A, left). Quantitation indicated that pro-BACE1 protein levels increased at 1 day (1.49 ± 0.47), then decreased swiftly at 3 day (0.53 ± 0.06) or 7 day (0.54 ± 0.22) after PBBI (Figure 5A, center). Mature BACE1 protein levels intensified at 3 day (0.18 ± 0.04) and 7 day (0.33 ± 0.20) (Figure 5A, right). In accordance with this observation, western blot analysis indicated that APP (∼100 kDa) was decreased after PBBI (Figure 5B, left). Here, APP protein levels fell precipitously at 3 day (0.13 ± 0.02) and 7 day (0.05 ± 0.03) after PBBI compared to Sham-operated controls (Figure 5B, right). APP loss was also detected per the use of the N-terminal antibody, 22C11 (data not shown). Secreted APP-α/β, C-terminal fragments, and Aβ peptides are main products of BACE1 and PSEN1/2 APP degradation. Thus, levels of these peptides was tested in brain tissue lysates. Cleavage products, such as secreted APP-α/β and C-terminal fragments (CTFs), were detectable in this study; but the resolution was poor (data not shown). However, Aβ-40 content increased after PBBI (0.43 ± 0.05 ng/mL) relative to Sham-operated controls (0.30 ± 0.06 ng/mL) at 7 day (Figure 5C, left). Surprisingly, Aβ-42 decreased, albeit marginally, after PBBI (0.05 ± 0.005 ng/mL) compared to Sham-operated (0.07 ± 0.003 ng/mL) at this same time-point (Figure 5C, center). Fold change transformation of the peptide data further illustrates the increase in Aβ-40 versus Aβ-42 at 7 day after PBBI (Figure 5C, right). Here, the peptide ratio doubled (9.1 ± 1.1 AU) compared to Sham-operated controls (4.5 ± 1.0 AU).

## Discussion

Analysis of miRNAs and their proposed targets can shed light upon TBI – induced processes that are associated with neurodegeneration. MiRNA array analysis, systems biology, and quantitation of downstream mRNA targets (inferred from systems biology analysis) indicated that a subset of miRNAs affected by PBBI were specific for each time-point and associated with inflammation or neurodegeneration, well-known aspects of TBI progression. Further, several miRNAs were specifically associated with mRNAs within the APP-processing pathway, particularly BACE1, a key APP degrading enzyme.

### PBBI Leads to Temporally Specific miRNA Dysregulation Associated With Inflammation and Neurodegeneration

Our earlier work indicated that miRNAs affected by PBBI were associated with inflammation (Johnson et al., 2017). It is likely that miRNAs are correlated with, and perhaps involved in, a myriad of deleterious processes that occur throughout TBI progression. The data presented herein collectively indicates that PBBI leads to transcriptional dysregulation of more than 60 miRNAs, most of which occurred 7 day after PBBI. In comparison, fluid percussion injury (FPI) has been reported to affect a set of 24–27 miRNAs 1–3 days post-injury (Lei et al., 2009). Ten miRNAs were consistently dysregulated in hippocampal lysates collected 1 h–7 day after controlled cortical impact (CCI), which was defined after comparison to a Sham-operated control collected at a single time-point after injury (Liu et al., 2014a).

Several of the miRNAs affected by PBBI in this study have been reported to be involved in inflammation as well as neurodegeneration. MiR-667 and miR-335 were both decreased 3–7 days after PBBI. MiR-667 is a transcriptional repressor of catechol-*O*-methyltransferase (COMT) (Segall et al., 2015), a gene that is upregulated in response to trauma-induced microglial activation (Redell and Dash, 2007). MiR-335 targets insulin-like growth factor receptor (IGF-R) (Gong et al., 2014). The IGF-1/IGF-R pathway is crucial to cellular metabolism affected by TBI and the ligand, IGF-1, is increased in damaged cortical and hippocampal regions 3–7 day after PBBI (Madathil and Saatman, 2015; Madathil et al., 2017). It is possible that this observation is relevant to neurogenesis during attempted repair (Madathil et al., 2013). However, the interaction between miRNA and gene targets that overlap processes, such as inflammation, neurodegeneration, and neurogenesis require further study within this model. PBBI-induced upregulation of miR-155 and -223 may occur as a consequence of microglial activation (Thounaojam et al., 2013; Lee et al., 2018) as well as post-traumatic mitochondrial injury, a novel therapeutic target for TBI (Wang et al., 2015; Harmon et al., 2017).

MiR-21 is one of the most well studied suppressors of gene expression in cellular biology and is considered a master regulator of cell growth and proliferation in response to post-traumatic apoptosis (Krichevsky and Gabriely, 2009). For instance, subacute upregulation of miR-21 reduces neuronal cell death and blood brain barrier damage, indicating that it may have a due role in cellular damage as well as repair after TBI that is dependent upon the time frame, brain region, or cell type affected (Ge et al., 2014, 2015; Han et al., 2014). Upregulation of miR-21 was previously found to be associated with pro-inflammatory cytokine upregulation in the PBBI and CCI models (Redell et al., 2011; Johnson et al., 2017). The relative influence that miR-21 may have in the presence of other miRNAs at various time-points is not known, upregulation in this study infers that miR-21 may have utility as a key regulator, or hub, within multiple TBI models.

Of note, this study used a database that annotates all interactions for “mmu-miR-21#” (the -3p strand) and “hsa-miR-21” (the -5p strand) into a single entry for miR-21. Although both sequences have been shown to be dysregulated in TBI models and we are aware that miR-21-3p and miR-21-5p may have opposing functions (Ge et al., 2019), we have chosen to validate miR-21-5p due to evidence of its upregulation in our previous study of the PBBI model, specifically (Johnson et al., 2017). The relative impact of -3p versus -5p sequences are considered in the context co-expressed biomarkers (Choo et al., 2014) or for therapeutic strategies involving inflammation. These topics are key, yet potentially broad, features of TBI progression that offer rationale to understand interactions and roles involving miRNA within concurrent and future studies. Instead, these data have also uncovered potentially unified processes associated with protein degradation.

### PBBI-Induced MiRNA Dysregulation Is Associated With BACE1 Upregulation and APP Loss Relevant to Widespread Degradation

Associative analysis of miRNAs is highly informative in regards to known events in TBI progression. Arrays coupled to pathway analysis may serve as predicates to further refine hypotheses and conduct secondary assessment. A minor caveat of all genetics studies and translation to protein levels is that the absolute fold change data derived from arrays occasionally varies from that of individual qPCR. This slight change is expected due to differences in RNA isolation and the limits of detection of platforms used, as well as biological variance inherent to animal models. A common, yet under-reported source of variance exists across different qPCR platforms such as open array and single tube or 96 well (Mestdagh et al., 2014; Farr et al., 2015; Martin-Alonso et al., 2018). Low abundant transcripts introduce a higher variability on high throughput platforms, even though pre-amplification steps are introduced. In PBBI, an acceptable range of variance is common among genes or proteins as shown in prior reports which is associated with ipsilateral lesion volume and tissue loss in any single cohort (Mondello et al., 2016). However, widespread protein and brain tissue degradation is a consistent feature of open skull, severe TBI models per observation of apoptosis (Brophy et al., 2009; Cartagena et al., 2013), as well as axonal fiber and cellular degeneration (Brophy et al., 2009; Shear et al., 2009; Gajavelli et al., 2015). A novel aspect of this model is presented by this study, which found that miRNAs affected by PBBI over time collectively mapped to APP processing, a key feature of neurodegeneration (Roberts et al., 1994; Nguyen, 2019; Shin et al., 2019), that overlaps the timeframe of widespread degradation in this model.

Although APP processing is dominated by non-amyloidogenic alpha (α)-secretase cleavage pathway involving ADAM family enzymes (Parvathy et al., 1999), the amyloidogenic mechanism of APP degradation involves two key enzymes, β-secretase (BACE1) and the gamma (γ)-secretase complex composed of PSEN1/2 (Vassar et al., 1999; Zhang et al., 2001; Chow et al., 2010) and other proteins which can be activated under pathological conditions (Zhang et al., 2011). In this case, APP is cleaved by β-secretase to release sAPPβ and CTF99, then the γ-secretase complex, cleaves CTF99 to generate Aβ-40/-42 (Cole and Vassar, 2008). Our lab previously indicated that APP loss occurs during subacute PBBI (Cartagena et al., 2016). Therefore, the results presented herein suggest that miRNAs may be coordinated with APP cleavage through enzymes during acute-subacute TBI progression.

This study showed miR-21 or -214 elevation coupled with miRNA-328 or -135A suppression were associated with BACE1 and PSEN2 modulation, which is in accordance with former studies detailing suppression of BACE1 by miR-135A, miR-328, and miR-29c in cell cultures (Zong et al., 2011; Liu et al., 2014b) and transgenic mouse models of AD (Boissonneault et al., 2009). In post-mortem brain tissues derived from patients diagnosed with non-familial AD, miR29c or miR-328 were inversely proportional to BACE1 gene levels (Hebert et al., 2008; Shioya et al., 2010; Lei et al., 2015). Increased levels of miR-223 inversely related to ADAM17 were observed. Yet, no miRNAs directly associated with ADAM10, and there were no relationships observed 3 day after PBBI. Loss of two, but upregulation of one, miRNA that each have negative relationships with BACE1 coupled with potential suppression of ADAM17 and the lack of a role for ADAM10, infer coordination of miRNA and mRNA events within injured brain tissue in line with a net gain of BACE1 as a key factor. Additional evaluation is on-going to determine the relative influence of ADAM10, and ADAM17 compared to BACE1 in this model.

Delayed upregulation and cleavage to the mature form of BACE1 during APP loss infers that injured brain tissue undergo proteolysis or remodeling throughout subacute time-frames. The relative increase in Aβ-40, compared to near ablation of full length APP, was somewhat small. Therefore, the role of BACE1 may be more robust than that of γ-secretase in this model. This observation is fitting for several reasons. First, BACE1 transcripts and β-secretase activity are upregulated in damaged neurons and activated astrocytes as a consequence of acute-subacute TBI (Blasko et al., 2004; Yu et al., 2012). BACE1 cleaves a variety of substrates (Hemming et al., 2009) and is associated with decreased dendritic spine integrity (Savonenko et al., 2008) and axonal blebbing (Hitt et al., 2012; Hu et al., 2015), which is due to an increase in the number of APP positive neuronal terminals (Tran et al., 2011) after brain trauma. This feature has been detected in the PBBI model upon detection of increased immunoreactivity from a C-terminal antibody that recognizes both APP and Aβ peptides (Lu et al., 2016). A large swath of the ipsilateral brain is damaged by PBBI. Thus, APP positive cells likely contain a mixture of intact as well as cleaved peptides (Burton et al., 2008; Wahrle et al., 2008; Muresan et al., 2009). The PBBI model does not replicate amyloidosis (plaques) *per se*, but it does lead to drastic fragmentation of many neuronal proteins (DeDominicis et al., 2018), inclusive of APP as previously mentioned, in addition to tissue loss in a manner that is much more drastic than CCI or FPI (Mountney et al., 2016).

Second, APP degradation may occur via multiple enzymatic pathways (Abrahamson et al., 2006; Ikonomovic et al., 2017) although it is mostly widely studied in the context of degenerative Aβ plaques (Hartig et al., 2010; Gouras et al., 2015). It is possible that PBBI leads to widespread APP fragmentation associated with miRNA dysregulation and BACE1, prior to redistribution or seeding of APP as seen in this model at later time points. This aspect of brain trauma may be similar to the observation that APP and its fragments are detectable in the soma, as well as dendritic and axonal terminals of injured cells (DeBoer et al., 2014; Niederst et al., 2015). Thus, the observed shifts in BACE1 and APP associated with this miRNA profile is a reflection of widespread damage to the brain tissue.

Lastly, the later steps in APP processing rely upon PSEN1/2 variants within the active γ-secretase complex may be responsible for extensive and sustained Aβ-40/42 peptide generation and seeding after action upon APP by BACE1. APP and its peptides are often studied in TBI rodent models that carry the mutated PSEN transgenes (Cheng et al., 2019) in an effort to show direct relationships to early onset dementia (Gotz et al., 2018). These mutations are rare and indicate that analysis in wild type models, as in this study, are increasingly important. Notably, Aβ-40 is the dominant peptide in brain trauma as well as sporadic (non-transgenic) AD in rat models (Shin et al., 1997). The negative relationship between miR-135A (decreased at 1 day) or miR-214 (increased, at 7 day) and either PSEN1 were sparse compared to the relationships shown for BACE1 over time. Further, the other γ-secretase complex proteins (e.g., nicastrin) (Kimberly et al., 2003) were not associated with the miRNAs expressed in PBBI damaged tissues found in this study. As such, Aβ-40 may be the expected dominant form of APP peptide produced in a TBI model.

A potential caveat of this study is that resolution of sAPPα or sAPPβ was poor in this model per the techniques used. Future work will explore use of alternative methods. Although APP degradation is well studied in cell culture models, post-mortem human brain, or transgenic mice in the context of AD, it remains relatively understudied brain tissues derived from wild-type rat models subjected to the most severe, survivable hemorrhagic model of TBI. It is also possible that the molar ratio of sAPPα or sAPPβ may be too low for detection by current techniques in this context. Assessment of these fragments is on-going for this model. Overall, miRNA associated BACE1 upregulation may be the key initial step in wholesale APP degradation after TBI.

## Conclusion

A subset of miRNAs found in this study may be putative to TBI-induced inflammation and neurodegeneration. These miRNAs, as well as BACE1, within the APP pathway may serve as novel biomarkers of degradation or reduced neuronal integrity in brain tissues. More importantly, this study is the first to show that coordinated miRNA dysregulation, elevation of BACE1, and post-transcriptional APP loss co-occur in a non-transgenic rodent model of severe TBI. The observations presented are associative, yet use of arrays and pathway analysis are useful in augmenting hypotheses to study the secondary effects of TBI that lead to widespread cell and tissue loss defined by discreet, rather than broad, analysis. MiRNAs regulate broad networks of genes. Thus, identifying those which are dysregulated as a consequence of TBI offer a means to elucidate molecular mechanisms, or hubs, that underlie neurodegeneration and may serve as viable therapeutic targets. Refinement of interaction mapping and connectivity displays per extensive knowledge of APP processing and studies to determine direct roles of these miRNAs, inclusive of *in vivo* interactions in the PBBI model, are in progress.

## Data Availability Statement

All datasets presented in this study are included in the article/Supplementary Material.

## Ethics Statement

The experimental procedures were approved by the WRAIR Animal Care and Use Committee. Research was conducted in compliance with the Animal Welfare Act and other federal statutes and regulations relating to animals and experiments involving animals, and adheres to principles stated in the Guide for the Care and Use of Laboratory Animals, NRC Publication, 2011 edition.

## Author Contributions

BT, DJ, and AMB conceived the experimental design and conducted the sample preparation and data analysis. All authors prepared the manuscript.

## Disclaimer

AMB is the owner/founder of Aries Biotechnologies (Oakland, CA, United States), which had no role in this work. Material has been reviewed by the Walter Reed Army Institute of Research. There is no objection to its presentation and/or publication. The opinions or assertions contained herein are the private views of the author, and are not to be construed as official, or as reflecting true views of the Department of the Army or the Department of Defense.

## Conflict of Interest

The authors declare that the research was conducted in the absence of any commercial or financial relationships that could be construed as a potential conflict of interest.

## Footnotes

1 http://bioinfogp.cnb.csic.es/tools/venny/
